# Characterization of a cytokinin-binding protein locus in *Mycobacterium tuberculosis*

**DOI:** 10.1128/jb.00003-25

**Published:** 2025-02-27

**Authors:** Jin Hee Yoo, Cristina Santarossa, Audrey Thomas, Damian Ekiert, K. Heran Darwin

**Affiliations:** 1 Department of Microbiology, New York University School of Medicine 12296 , New York, New York, USA; 2 Department of Cell Biology, New York University School of Medicine 12296 , New York, New York, USA; University of Notre Dame, Notre Dame, Indiana, USA

**Keywords:** mycobacteria, cytokinins

## Abstract

Cytokinins are adenine-based hormones that have been well-characterized in plants but are also made by bacteria, including the human-exclusive pathogen *Mycobacterium tuberculosis*. Like plants, *M. tuberculosis* uses cytokinins to regulate gene expression. We previously established that cytokinin overaccumulation in *M. tuberculosis* results in a buildup of aldehydes produced during cytokinin breakdown. In plants, dedicated enzymes called cytokinin oxidases convert cytokinins into adenine and various aldehydes. Proteasome degradation-deficient *M. tuberculosis*, which cannot degrade the cytokinin-producing enzyme Log, accumulates several cytokinins and at least one cytokinin-associated aldehyde, resulting in increased sensitivity to nitric oxide and copper. We therefore hypothesized that *M. tuberculosis* encodes one or more cytokinin oxidases, and disruption of this enzyme might restore resistance to nitric oxide and copper in a proteasome-defective strain. Using a homology-based search, we identified Rv3719 as a protein with high similarity to a plant cytokinin oxidase. Deletion of this gene, however, did not restore nitric oxide or copper resistance to a degradation-defective mutant. Instead, we observed increased copper sensitivity when Rv3719 was deleted from either wild-type or proteasome-defective strains. Finally, we characterized Rv3718c, a protein encoded adjacent to Rv3719, and found that it bound a cytokinin with high specificity. Collectively, these data support a role for cytokinin activity in *M. tuberculosis* physiology that remains to be further elucidated.

**IMPORTANCE:**

Numerous bacterial species encode cytokinin-producing enzymes, the functions of which are almost completely unknown. This work contributes new knowledge to the cytokinin field for bacteria and reveals further conservation of cytokinin-associated proteins between plants and prokaryotes.

## INTRODUCTION

*Mycobacterium tuberculosis* is estimated to be present in almost one-third of the world’s population. Given that the existing vaccine does not provide reliable protection and drug treatment is prolonged or ineffective in drug-resistant strains, there are ongoing efforts to identify new pathways to pursue tuberculosis treatment. A major target of interest is the *M. tuberculosis* proteasome, which is essential to cause lethal infections in mice (1, 2). Proteasomes are highly regulated, multi-subunit, ATP-dependent proteases found in all domains of life. *M. tuberculosis* proteasomes degrade numerous substrates with significant roles in bacterial physiology (reviewed in reference [3]). Many substrates must first be posttranslationally modified with the protein Pup that targets doomed proteins to an ATP-dependent hexameric ring called Mpa (mycobacterial proteasome ATPase, known as ARC in non-mycobacteria), which unfolds substrates to deliver them into a proteasome core protease. Previous studies determined that the failed degradation of a single pupylated substrate, “lonely guy” (Log or Rv1205), results in bacteria that are highly sensitive to nitric oxide (NO) (4) and copper (Cu) (5).

Log was first characterized in plants, and homologs are now well-recognized in numerous bacterial and fungal species. In both plants and *M. tuberculosis*, Log catalyzes the final step in cytokinin biosynthesis (4, 6). Cytokinins, which are adenine-based hormones with a modification at the nitrogen (*N*)^6^ position of the adenine rings, have been extensively studied in plants and are required for their normal growth and development (7, 8). In *M. tuberculosis*, cytokinins induce transcription of *loaA* (Rv0077c), resulting in a dramatic loss of acid-fast staining of bacteria (9). *loaA* is divergently expressed from *loaR*, which encodes a TetR-like repressor that represses *loaA*. In the presence of cytokinins, *loaA* expression is induced, presumably by the release of LoaR from its promoter. Because TetR-type repressors generally bind a small-molecule ligand to relieve repression, it was initially assumed that LoaR binds to one or more cytokinins; however, LoaR does not bind to cytokinin (9). Thus, how cytokinins induce LoaR derepression of *loaA* is unknown. Moreover, how LoaA results in the loss of acid-fast staining remains to be determined.

In plants, cytokinins are enzymatically degraded into adenine and aldehydes, presumably to abate hormone signaling and recycle their components. Adenine can be easily incorporated into numerous molecules, while aldehydes likely react with other macromolecules or are detoxified. In an *mpa* mutant, the accumulation of Log results in high levels of several secreted cytokinins and at least one cytokinin-associated aldehyde called *para*-hydroxybenzaldehyde (*p*HBA), a breakdown product of *p*-topolin (10). Disruption of *log* is sufficient to reduce *p*HBA levels and fully restore NO and Cu resistance to an *mpa* mutant (4, 5). Importantly, exogenous *p*HBA is sufficient to sensitize wild-type (WT) *M. tuberculosis* to NO and Cu, suggesting that it is *p*HBA that disrupts resistance to these molecules (4, 5).

Cytokinin oxidases or dehydrogenases (CKX) are enzymes in plants that break down cytokinins by removing chemical groups from the *N^6^* position of their adenine rings, liberating the associated aldehydes (10). Moreover, it is unlikely that cytokinins spontaneously break down. We thus hypothesized that *M. tuberculosis* encodes a CKX needed to break down one or more cytokinins and that disrupting a CKX-encoding gene would suppress the NO- or Cu-sensitive phenotypes of a proteasomal mutant, much like a *log* mutation suppresses sensitivity in an *mpa* mutant (4, 5). In this work, we identified an open reading frame, Rv3719, that encodes a protein with high similarity to a CKX from maize but found that disrupting this gene did not suppress the NO or Cu sensitivity of an *mpa* mutant. Unexpectedly, deletion of Rv3719 increased Cu sensitivity of both WT and *mpa* strains. Additionally, we determined that Rv3718c, which is divergently expressed from Rv3719, encodes a cytokinin-binding protein similar to ones found in plants. Thus, our work has identified the first cytokinin-binding protein in bacteria and a gene required for robust Cu resistance of *M. tuberculosis*.

## RESULTS AND DISCUSSION

### *M. tuberculosis* encodes putative cytokinin-associated proteins

**Fig 1:**
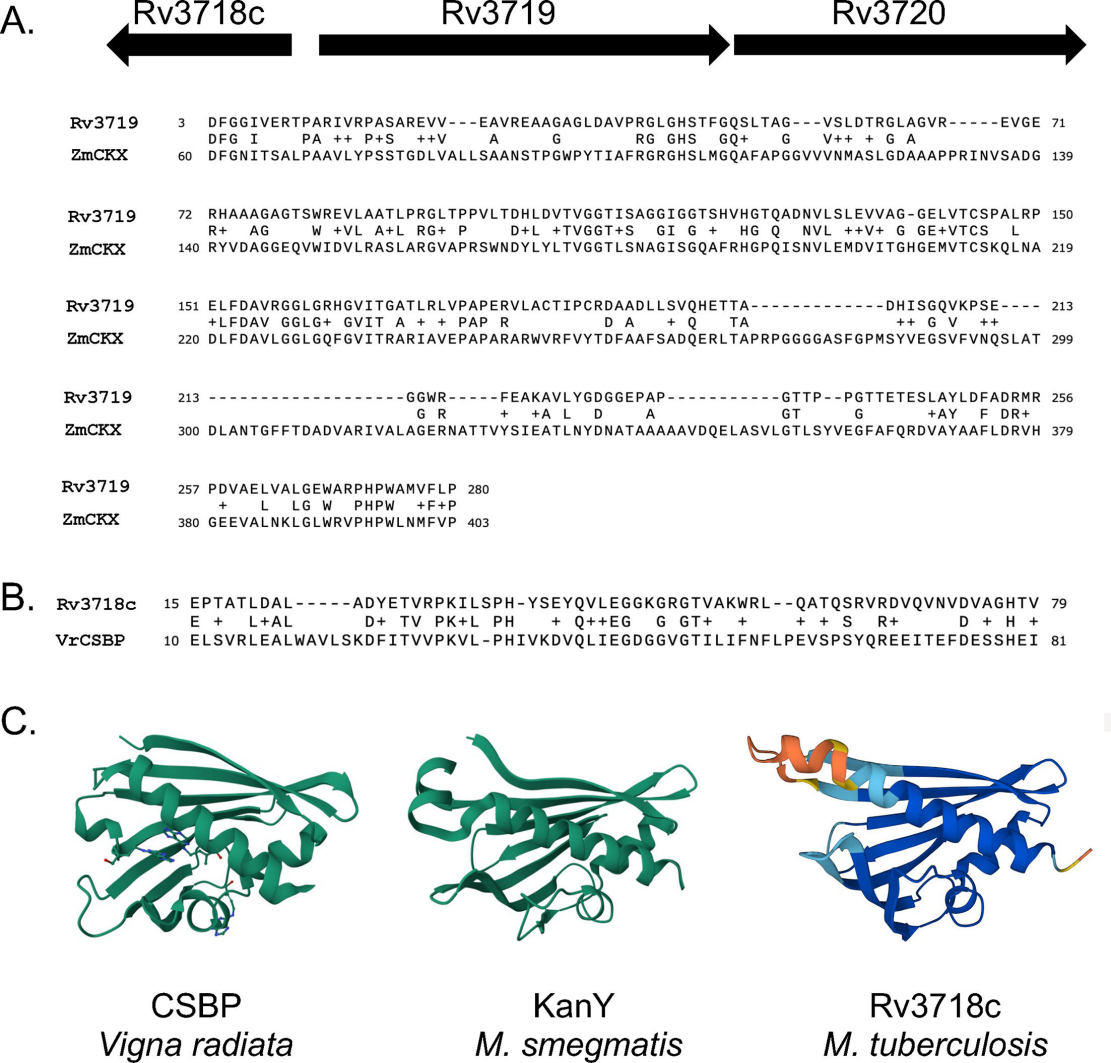
Putative cytokinin-binding proteins in *M. tuberculosis*. (**A**) Top: Map of Rv3718c-Rv3719-3720 locus in *M. tuberculosis*. Bottom: Alignment of *Zea mays* CKX with *M. tuberculosis* H37Rv Rv3719. (**B**) Alignment of *Vigna radiata* (Vr) CSBP with Rv3718c. (**C**) Comparison of VrCSBP (PDB: 2FLH) with *M. smegmatis* KanY (PDB: 5WOX) and the AlphaFold model of Rv3718c. Molecules in VrCSBP are the cytokinin zeatin.

We tested the hypothesis that *M. tuberculosis* has a CKX protein by performing a BLASTP search (11) using the characterized *Zea mays* (maize, corn) cytokinin dehydrogenase 1 (accession number Q9T0N8.2) against the *M. tuberculosis* proteome. The protein with the highest similarity (49/160, 30%) was Rv3719 (Fig. 1A). Despite numerous efforts, including the use of different epitope tags and bacterial expression systems, we could not produce recombinant Rv3719. Consistent with this inability to produce protein, we found using ProtParam (12) that Rv3719 is predicted to be unstable with an instability index of 48.04.

Downstream of Rv3719 is Rv3720, which is predicted to encode a fatty acid synthase. Given that some cytokinins have prenyl groups, it is possible that Rv3720 binds to one or more of these hydrophobic molecules for their metabolism. Divergently expressed from Rv3719 is Rv3718c (Fig. 1A), which is annotated as *kanY* in *M. smegmatis* for unknown reasons (accession number STZ35122). Rv3718c has high similarity with a cytokinin-specific binding protein (CSBP) from *Vigna radiata* (mung bean) (Fig. 1B). Interestingly, all three genes are conserved in *M. smegmatis* and *M. leprae*, the latter of which has a highly decayed genome (13). This observation suggests that this locus plays a core function in mycobacterial physiology. Comparison of either the nuclear magnetic resonance structure of MSMEG_6282 (KanY) or an AlphaFold model of Rv3718c (14) to CSBP revealed strong structural similarities among the proteins (Fig. 1C).

### Deletion of the Rv3718c-Rv3720 locus did not affect growth under routine culture conditions or expression of a cytokinin-inducible gene, *loaA*

**Fig 2:**
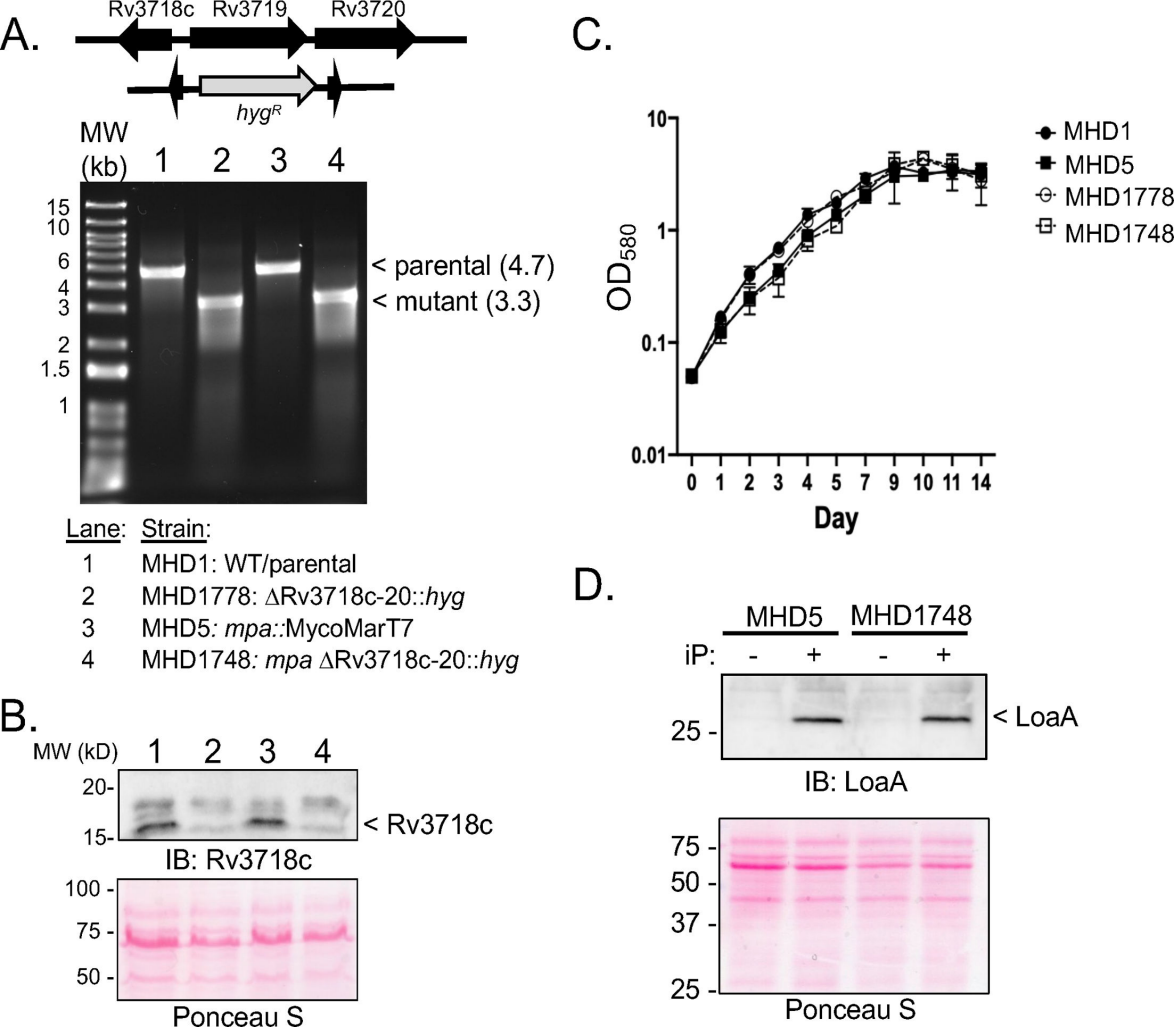
Deletion of Rv3718c-Rv3720 did not affect growth under routine culture conditions and LoaA production. (**A**) Schematic of the deleted region (top). Ethidium bromide-stained gel with PCR products from the indicated strains (bottom). Primers annealing outside of the region included in the allelic exchange vector were used (Rv3718-3720delF and Rv3718-3720delR ). Sizes in kilobases (kb) of expected PCR products are indicated in parentheses. (**B**) Rv3718c was undetectable in total cell lysates of strains lacking the Rv3718c-Rv3720 locus. IB, immunoblot. Ponceau S staining of the membranes showed even loading. Lanes are for the same strains indicated in panel **A**. (**C**) Deletion of Rv3718c-Rv3720 did not affect growth in Middlebrook 7H9c broth. Growth curves were performed in duplicate and are representative of two independent experiments. (**D**) Induction of the cytokinin-inducible gene, *loaA*, as detected by immunoblotting for LoaA. MHD5, *mpa*::MycoMarT7; MHD1748, *mpa*::MycoMarT7 ΔRv3718c-Rv3720. iP = 100 µM isopentenyladenine.

We simultaneously deleted and replaced all three genes with a hygromycin resistance cassette in the *M. tuberculosis* chromosome (Fig. 2A**,** top) with the expectation that their removal would increase the chance of a phenotype. We deleted these genes from both WT and *mpa M. tuberculosis* strains and confirmed the loss of the locus by PCR (Fig. 2A**,** lower panel) and Rv3718c protein by immunoblotting with polyclonal antibodies for Rv3718c_His_6_ (see Materials and Methods; Fig. 2B). Deletion of this locus had no measurable impact on growth under routine culture conditions (Fig. 2C).

In *M. tuberculosis*, cytokinins induce expression of *loaA* (Rv0077c), the product of which can be detected by immunoblotting (9). We hypothesized that a putative cytokinin-binding protein or cytokinin oxidase could affect cytokinin-dependent induction of *loaA*. Although an *mpa* mutant produces more cytokinins than a WT strain (15), it was not enough to auto-induce LoaA production (Fig. 2D, leftmost lane). Like a WT strain (15), the *mpa* mutant strongly produced LoaA in the presence of the cytokinin *N*^6^-(2-isopentenyl)adenine (also known as isopentenyladenine or iP) (Fig. 2D). Deletion of Rv3718c-Rv3720 from this strain did not alter LoaA production (Fig. 2D). Thus, the Rv3718c-Rv3720 locus does not appear to regulate cytokinin inducibility of *loaA* expression under the conditions tested.

### Deletion of Rv3719 resulted in Cu sensitivity

**Fig 3:**
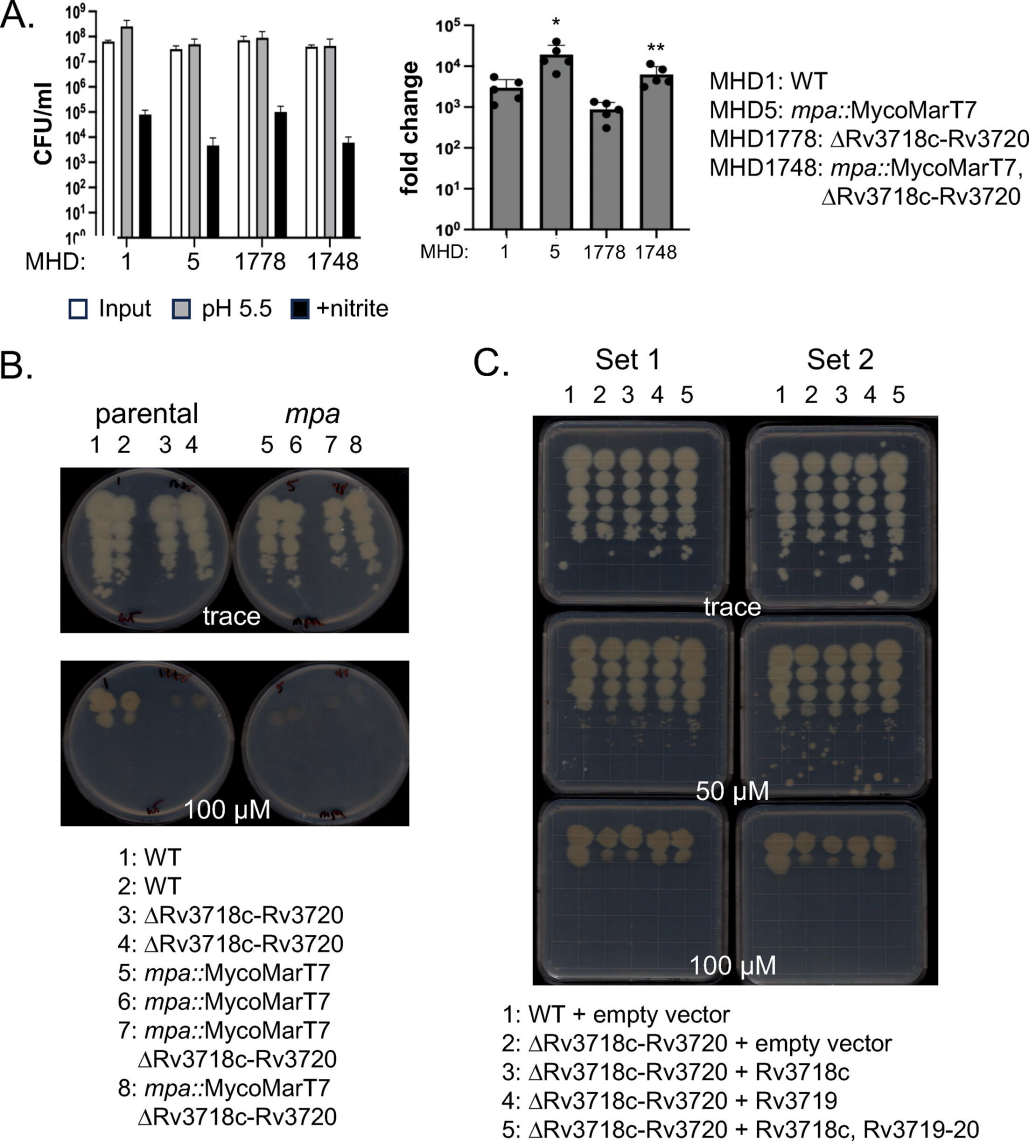
Deletion of Rv3718c-Rv3720 locus had WT NO resistance and a subtle defect in copper resistance. (**A**) Deletion of Rv3718c-Rv3720 did not affect sensitivity to NO. NO assays were performed in technical triplicate and are representative of two independent experiments with an outlier technical replicate omitted. Given the significant difference in growth between the parental and *mpa* strains without NO in these assays, we calculated the fold change in survival in NO compared to acidic media alone, which more accurately represents the susceptibility of the strains. * indicates *P* = 0.02; ** indicates *P =* 0.008. (**B**) Cu sensitivity assay. Equal CFU based on culture OD_580_ were inoculated onto the indicated agar plates and incubated for 19 days before imaging. “Parental” indicates WT H37Rv and its isogenic Rv3718c-Rv3720 deletion strain. “*mpa*” indicates a transposon insertion mutant strain and its isogenic Rv3718c-Rv3720 deletion strain. (**C**) Rv3719 partially complements Cu sensitivity. “Set” indicates independent experiments of two different isolates from each transformation.

We hypothesized that disrupting a putative CKX would reduce cytokinin breakdown and, consequently, reduce aldehyde production in a proteasomal degradation mutant, thereby restoring NO and Cu resistance. However, deletion of this locus did not suppress NO (Fig. 3A) or Cu (Fig. 3B) sensitivity. Instead, we observed a subtle but reproducible Cu hypersensitivity of the deletion strains (Fig. 3B, columns 3, 4, 7, and 8). We complemented the deletion mutation in the *mpa*^+^ background with Rv3718c, Rv3719, or all three genes. The entire locus or just Rv3719 resulted in partial complementation (Fig. 3C, columns 4 and 5 in both sets compared to columns 2 and 3). These data suggest that Rv3719 is required for WT Cu resistance.

It is possible that deleting Rv3719 resulted in a compensatory induction of one or more genes that perform a similar function. *M. tuberculosis* has at least three other gene products with similarity to *Z. mays* CKX (Rv0063, Rv1726, Rv1774). Future transcriptional analysis may reveal if one or more of these is induced in the absence of Rv3719 to “overcompensate” and result in Cu sensitivity due to aldehyde production. However, because NO sensitivity was unchanged in the absence of Rv3719, it seems less likely that aldehyde accumulation is responsible for the Cu-sensitive phenotype.

### Rv3718c is a cytokinin-specific binding protein

**Fig 4:**
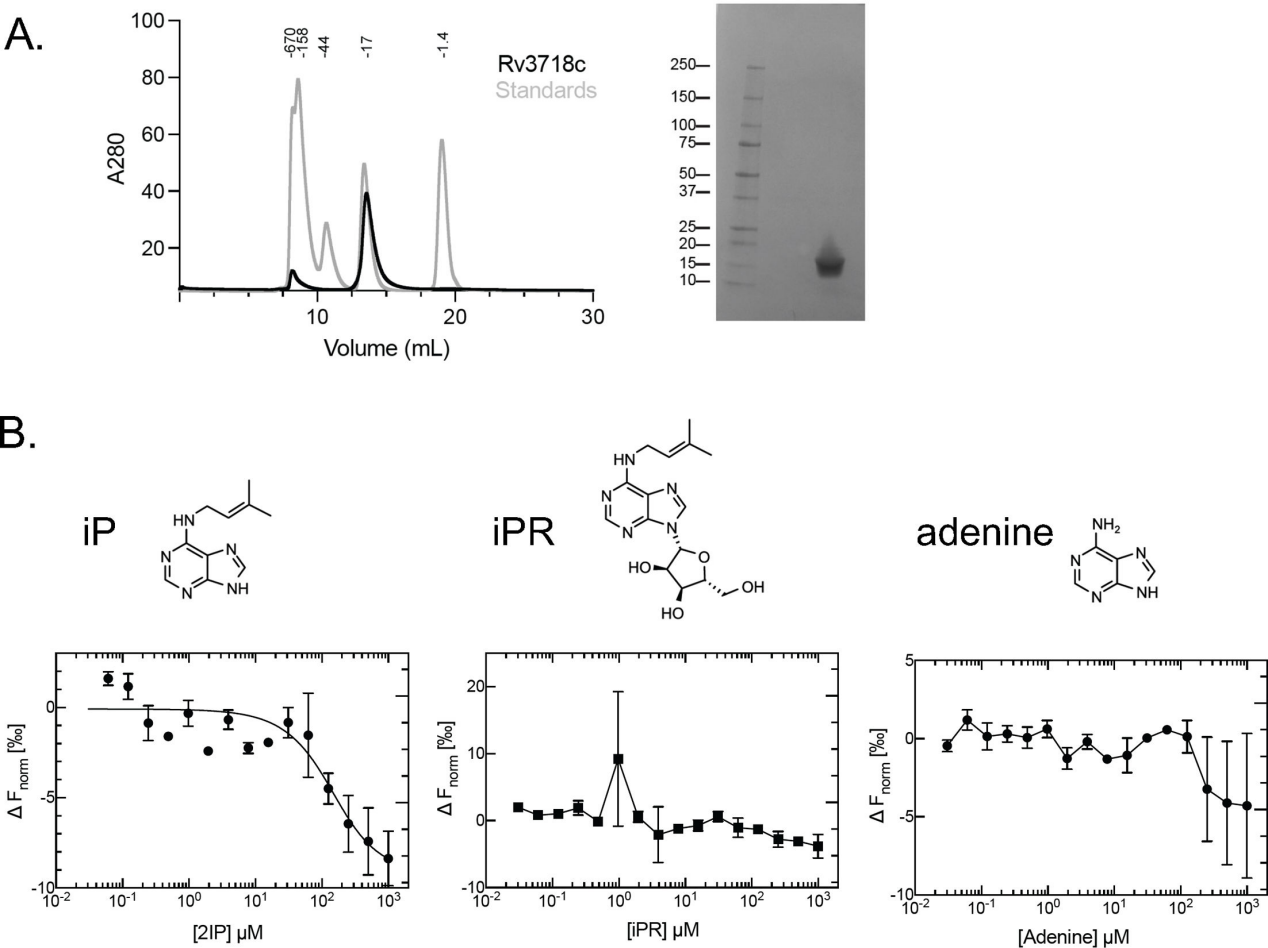
Rv3718c is a cytokinin-specific binding protein. Purification of Rv3718 and characterization of cytokinin binding. (**A**) Right: Size exclusion chromatography of Rv3718c-His6 (black) overlaid with molecular weight standards (gray). Left: SDS-PAGE gel of purified Rv3718c-His6. (**B**) Microscale thermophoresis (MST) analysis to assess binding of the indicated compounds (above) to Rv3718c-His_6_. Data are shown as mean ± range and were fit using equation 1. The individual data points are shown from two independent experiment.

To test if Rv3718c could bind the cytokinin iP or related molecules, we used microscale thermophoresis (MST). We purified C-terminally, hexahistidine-tagged Rv3718c from *E. coli* (Fig. 4A; see Materials and Methods) and found Rv3718c bound iP with an estimated K_d_ of ~125–250 µM (Fig. 4B). At the concentrations tested, Rv3718c did not bind *N*^6^-(Δ^2^-isopentenyl)adenosine (iPR), the riboside precursor of iP, or adenine, a precursor and breakdown product of iP (Fig. 4B**,** far right). These data suggest that Rv3718c is a cytokinin-specific binding protein. Given the structural and functional similarities with plant CSBP, we suggest naming the mycobacterial protein Cbp for “cytokinin-specific binding protein.”

Our hypothesis was to test if the inactivation of a putative CKX, Rv3719, could provide protection against NO or Cu. While failed degradation of the cytokinin activating enzyme Log weakens *M. tuberculosis* in NO, Cu, and mice (4, 5), the genes in the *cbp*-Rv3719-3720 locus were not essential for *M. tuberculosis* NO resistance (this work) or pathogenesis in at least one infection model (16). Nonetheless, the conservation of cytokinin signaling genes in mycobacteria, including *M. leprae*, strongly suggests that they play an important function in physiology at some specific point of a *Mycobacterium* life cycle, such as during infection of animals or protozoans. In addition to understanding the function of cytokinins in bacterial physiology, it remains to be determined how cytokinins transduce signals to affect gene expression. Plants use two-component regulatory systems (TCSs) closely related to bacterial TCSs. Although *M. tuberculosis* encodes at least 16 TCSs, the only transcription factor linked to cytokinin-inducible gene expression is LoaR, which does not bind cytokinin, and its native ligand has yet to be identified (9). Moreover, the present work did not find a link between *cbp*-Rv3719-3720 locus and *loaA* expression. Finally, because plant CSBP activities have not been determined, it is possible that the characterization of bacterial Cbps might help one day elucidate the role of these proteins in plant biology.

## MATERIALS AND METHODS

### Strains, plasmids, primers, and culture conditions

See Table 1 for strains, plasmids, and primers used in this work. Reagents used for making all buffers and bacterial media were purchased from Thermo Fisher Scientific, unless otherwise indicated. *M. tuberculosis* was grown in “7H9c“ (BD Difco Middlebrook 7H9 broth with 0.2% glycerol and supplemented with 0.5% bovine serum albumin [BSA], 0.2% dextrose, 0.085% sodium chloride, and 0.05% Tween-80). For solid media, *M. tuberculosis* was grown on Middlebrook 7H11 agar (“7H11,” BD Difco) containing 0.5% glycerol and supplemented with 10% final volume of BBL Middlebrook OADC Enrichment. For the selection of *M. tuberculosis*, the following antibiotics were used as needed: kanamycin 50 µg/mL and hygromycin 50 µg/mL. *E. coli* was cultured in Luria-Bertani (LB) broth or on LB agar (both BD Difco). Media were supplemented with the following antibiotics as needed: kanamycin 100 µg/mL and hygromycin 150 µg/mL. Cytokinin *N*^6^-(2-isopentenyl)adenine was purchased from Sigma-Aldrich (Catalog #D7674), adenine was purchased from Sigma (Catalog #A8626), and *N*^6^-(Δ^2^-isopentenyl)adenosine was purchased from Cayman Chemical Company (Catalog #20522).

**TABLE 1:** Bacterial strains, plasmids, and primers used in this work

***E. coli***	Relevant genotype	Source or reference
DH5α	F-, θ80ΔlacZM15 Δ(lacZYA-argF)U169 *deoR recA1 endA1*	Gibco, BRL
	*hsdR17* (r_k_-m_k_+) *phoA supE44* λ- *thi-1 gyrA96 relA1*	
BL21(DE3)	F- *ompT gal dcm lon hsdS*_*B*_ (r_B_- mB-)	New England Biolabs
	λ(DE3 [*lacI lacUV5-T7p07 ind1 sam7 nin5*] (*malB*^+^)_K-12_(λS)	
*M. tuberculosis*		
MHD1	H37Rv; wild type, American Type Culture Collection* #25618	*ATCC
MHD5	Kan^R^; *mpa*::MycoMarT7	(1)
MHD1778	Hyg^R^; ΔRv3718c-3720::hyg	This study
MHD1748	Kan^R^, Hyg^R^; *mpa*::MycoMarT7 ΔRv3718c-3720::*hyg*	This study
MHD1936	Hyg^R,^ Strep^R^; ΔRv3718c-3720::*hyg* pMV306.Strep	This study
MHD1937	Hyg^R,^ Strep^R^; ΔRv3718c-3720::*hyg* pMV-Rv3718c	This study
MHD1938	Hyg^R,^ Strep^R^; ΔRv3718c-3720::*hyg* pMV-Rv3719	This study
MHD1939	Hyg^R,^ Strep^R^; ΔRv3718c-3720::*hyg* pMV-all	This study
Plasmids		
pYUB854	Hyg^R^; allelic exchange vector	(17)
pYUB-ΔRv3718c-3720	Hyg^R^; for deletion and disruption of the Rv3718c-3720 locus	This study
pET24b(+)	Kan^R^; for IPTG-inducible production of recombinant proteins in *E. coli*	Novagen
pET24b(+)Rv3718c	Kan^R^; for IPTG-inducible production of Rv3718c-His_6_	This study
pMV306.Strep	Strep^R^; integrative vector, inserts into the L5 *attB* site	(4)
pMV-Rv3718c	Strep^R^; pMV306.Strep with Rv3718c	This study
pMV-Rv3719	Strep^R^; pMV306.Strep with Rv3719	This study
pMV-all	Strep^R^; pMV306.Strep with Rv3718c-Rv3719-Rv3720	This study
Primers	Sequence (5′ to 3′)	
SphI_3718cF	AGGCATGCTGACGACGATGCGGGCGCGGTGGGGGTG	
XbaI_3718cR	GCTCTAGAACATCGCCGGCATGGTCGTCTTCATCG	
NheI_3720F	TCGCTAGCCCCTAGGCGTTGTCTATCCGGCGCGCGCCC	
HindIII_3720R	CGAAGCTTGTGCGACCGCGCCGCGTCTGCTGCTGG	
Rv3718-3720delF	GGTTCGCCATGATAGTCGGGGTACT	
Rv3718-3720delR	CGGGCGGGCGACCCTGACCCGATAT	
NdeIRv3718cF	GTACCATATGGGACAGGTGAGCGCAGCCAGCA	
HindIIIRv3718chisR	GTACAAGCTTTCAATGATGATGATGATGATGGGCGTCACCCTCGAGTTCGGTCTTG	
OHD451_Rv3718MfeIF	TTACAATTGTCAGGCGTCACCCTCGAGTTCGGTC	
OHD453_Rv3719endF	ACTCCTTCTATACCCGCGAGGAGTT	
OHD452_Rv3719endR	GCTTTCTTCACAGTGTTGTAAGTCT	
OHD454_3720R_H3	CATAAGCTTCTAGGGCTGCCACCAGGGCCGCAGT	

To make the pYUBΔRv3718c-Rv3720 allelic exchange plasmid, 700 bp upstream of Rv3718c and downstream of Rv3720 were amplified by PCR and cloned into pYUB854 flanking a hygromycin resistance cassette (see Table 1 for primers). For pET24b(+)-Rv3718c, primers NdeIRv3718cF and HindIIIRv3718chisR were used to PCR amplify Rv3718c from genomic DNA. For making the complementation plasmids, we used pMV306.Strep and the primers listed in Table 1. *E. coli* DH5α was used for transformations. Plasmids were purified from *E. coli* using the QIAprep Spin Miniprep Kit (Qiagen). All plasmids made by PCR cloning were sequenced by Azenta, Inc. to ensure the veracity of the cloned sequences. Primers used for PCR amplification or sequencing were purchased from Life Technologies or IDT and are listed in Table 1. DNA was PCR-amplified using Phusion polymerase (New England Biolabs, NEB) according to the manufacturer’s instructions. PCR products were purified using the QIAquick Gel Extraction Kit (Qiagen). Restriction enzymes and T4 DNA ligase were purchased from NEB.

*M. tuberculosis* was transformed by electroporation as previously described (18). For allelic exchange, we used a previously described method (15). Chromosomal DNA was purified from transformants and screened by PCR using primers Rv3718-3720delF and Rv3718-3720delR to determine if the locus was lost from transformants.

All *M. tuberculosis* work was performed in the ABSL3 facility of NYU Grossman School of Medicine, in accordance with its Biosafety Manual and Standard Operating Procedures.

### Purification and labeling of Rv3718c

Hexahistidine-tagged Rv3718c was produced in *Escherichia coli* BL21(DE3) cells and grown in LB at 37°C. Expression was induced at OD_600_ 0.6–0.7 with 0.1 mM isopropyl B-D-1-thiogalactopyranoside (IPTG) and grown for 4 hours at 37°C. Bacteria were pelleted and resuspended in buffer containing 50 mM NaH_2_PO_4_ pH 8.0, 100 mM NaCl, and 10 mM imidazole. All subsequent purification steps were performed at 4°C. Cells were lysed using the Emulsiflex-C5 homogenizer (Avestin, 2 cycles at 15,000  psi), and the homogenized lysate was centrifuged at 30,000 *g* for 1 hour. The clarified lysate was applied onto a column containing Ni Sepharose 6 resin (Cytiva Life Sciences) and washed with 20 column volumes of buffer containing 50 mM NaH_2_PO_4_ pH 8.0, 100 mM NaCl, and 20 mM imidazole. The protein was eluted with buffer containing 50 mM NaH_2_PO_4_ pH 8.0, 100 mM NaCl, and 250 mM imidazole. The eluent was concentrated using an Amicon Ultra 3 kD MWCO centrifugal filter and then exchanged into size-exclusion buffer (20 mM HEPES pH 8.0, 100 mM NaCl, 5% glycerol) using Zeba Spin Desalting Columns (MW cutoff 7 kD, 0.5 mL). Rv3718c was labeled with Alexa Fluor 647 NHS Ester (Invitrogen Catalog# A20106) for 30 minutes at room temperature in the dark. The sample was loaded onto a Superdex 75 Increase 10/300 Gl column (Cytiva Life Sciences) in size-exclusion buffer. Fractions were pooled, yielding protein concentrations of 8–14 µM, flash frozen in liquid nitrogen, and stored at −80°C.

### Antibody production

We purified Rv3718c as described above, buffer exchanged it into PBS, and shipped ~1 mg of protein to Covance Laboratories, Inc. (Denver, PA) for injection into rabbits with Freund’s incomplete adjuvant.

### Microscale thermophoresis assay

The binding affinities of iP and iPR to Rv3718c were measured using Monolith NT.115 (NanoTemper Technologies, Munich, Germany). Frozen protein aliquots were thawed and diluted in MST buffer (20 mM HEPES pH 8.0, 100 mM NaCl, 5% glycerol, and 0.005% Tween 20). To obtain a fluorescence intensity between 200 and 1,100 counts at 20% LED power, a final concentration of 100 nM protein was used. A 25X compound stock was prepared by a 1:1 serial dilution of the compound in DMSO starting from 25 mM and then diluted in MST buffer to obtain a 2X compound stock. The 2X protein stock (200 nM Rv3718c) was mixed well with 2X compound and incubated for 5 minutes at RT in the dark. The final DMSO concentration is 4%. Each sample was transferred to a Monolith premium capillary tube, and measurements were performed at 20, 40, and 60% MST power at 25°C. At 60% MST power, a binding curve for iP was observed at 10 seconds after the start of thermophoresis. The ΔF_norm_ values were fitted to equation (1) using Prism v. 10.1.1 (GraphPad Software Inc) at different concentrations of 2iP.


(1) $$f\left(c_{ligand}\right)=Unbound+\left(Bound-Unbound\right)\times \frac{c_{ligand}+c_{target}+K_{d}-\sqrt{(c_{ligand}+c_{target}+K_{d}{)}^{2}-4\times c_{ligand}\times c_{target}}}{2c_{target}}$$

In equation (1), *f(c_ligand_)* is the *F*_norm_ value at a given ligand concentration *c_ligand_*, *Unbound* is the *F*_norm_ signal of the target alone, *Bound* is the *F*_norm_ signal of the complex, *K_d_* is the dissociation constant or binding affinity, and *c_target_* is the final concentration of the target.

### Preparation of *M. tuberculosis* extracts for immunoblotting

*M. tuberculosis* cultures were grown to an optical density at 580 nm (OD_580_) of ~1. Equivalent cell numbers were collected based on the OD_580_ of the cultures. For example, an “OD_580_ equivalent of 1” indicates that the OD_580_ of a 1 mL culture is 1.0. Five OD_580_ equivalents of bacteria were harvested by centrifugation at 3,000 *g,* washed in PBST (PBS, 0.05% Tween 80), resuspended in lysis buffer (100 mM Tris-Cl pH8, 1 mM EDTA pH8), and transferred to a tube containing 250 µL of 0.1 mm zirconia beads (BioSpec Products). Bacteria were lysed using a mechanical bead beater (BioSpec Products). Whole lysates were mixed with 4 × reducing SDS sample buffer (250 mM Tris pH 6.8, 2% SDS, 20% 2-mercaptoethanol, 40% glycerol, 1% bromophenol blue) to a 1× final concentration, and samples were boiled for 10 minutes at 100°C.

For immunoblotting, whole cell lysates were separated by 10% sodium dodecyl sulfate-polyacrylamide gel electrophoresis (SDS-PAGE). Proteins were transferred to nitrocellulose membranes (GE Amersham) by semidry transfer for 10 minutes at 15V (Bio-Rad) and stained with Ponceau S and scanned. After rinsing off the stain with water, blots were air-dried followed by blocking in 3% BSA in TBST and incubation with primary antiserum. Horseradish peroxidase-conjugated secondary antibodies to rabbit IgG were purchased from Pierce and developed using SuperSignal West Pico PLUS chemiluminescent substrate (Thermo Fisher Scientific) and imaged using the Bio-Rad ChemiDoc system and quantified using Fiji (19).

### Nitric oxide sensitivity assay

Assays were performed as described previously (1). Briefly, bacteria were grown to an OD_580_ ~0.8–1 and resuspended in acidified 7H9c (pH 5.5) and declumped by a slow-speed spin (800 *g*). The supernatant was then diluted to an OD_580_ of 0.08. Bacteria were then aliquoted in triplicate to flat-bottom 96-well plates, and a fresh sodium nitrite solution was added to each well at a final concentration of 3 mM. Bacteria were incubated for 6 days at 37℃ before plating onto 7H11 OADC Y plates and incubated at 37℃ for enumeration 2–3 weeks later. Data were graphed and analyzed in Prism, with *P* values calculated using parametric, unpaired *t*-tests.

### Copper sensitivity assay

Assays were done as previously described (20). Briefly, Middlebrook 7H11 agar plates were supplemented with CuSO_4_ at the indicated concentrations. Bacteria were grown to an OD_580_ ~1, collected by centrifugation, and resuspended in phosphate-buffered saline with 0.05% Tween 80. Bacteria were declumped by a slow-speed spin (800 *g*) and diluted to OD_580_ 0.01. This suspension was then serially diluted 10-fold, six times. Agar plates were prewarmed to 37℃ overnight before 3 µL of diluted bacteria were spotted onto the plates. Plates were incubated at 37℃ for 18–20 days before imaging.

## Data Availability

All data supporting these findings are available within the article.
